# N-Lactoyl Phenylalanine Disrupts Insulin Signaling, Induces Inflammation, and Impairs Mitochondrial Respiration in Cell Models

**DOI:** 10.3390/cells14161296

**Published:** 2025-08-20

**Authors:** Laila Hedaya, Khaled Naja, Shamma Almuraikhy, Najeha Anwardeen, Asma A. Elashi, Maha Al-Asmakh, Susu M. Zughaier, Meritxell Espino-Guarch, Osama Y. Aldirbashi, Gavin P. Davey, Mohamed A. Elrayess

**Affiliations:** 1 Biomedical Research Center, QU Health, Qatar University, Doha P.O. Box 2713, Qatar; laila.hedaya@qu.edu.qa (L.H.); khaled.naja@qu.edu.qa (K.N.); salmuraikhy@qu.edu.qa (S.A.); n.anwardeen@qu.edu.qa (N.A.); asma.elashi@qu.edu.qa (A.A.E.); 2 Department of Biomedical Sciences, College of Health Sciences, QU Health, Qatar University, Doha P.O. Box 2713, Qatar; maha.alasmakh@qu.edu.qa; 3 College of Medicine, QU Health, Qatar University, Doha P.O. Box 2713, Qatar; szughaier@qu.edu.qa; 4 Laboratory of Immunoregulation, Translational Medicine, Sidra Medicine, Doha P.O. Box 26999, Qatar; mespinoguarch@sidra.org; 5 Department of Lab Medicine & Pathology, Hamad Medical Corporation, Doha P.O. Box 3050, Qatar; oaldirbashi@hamad.qa; 6 College of Health Sciences, QU Health, Qatar University, Doha P.O. Box 2713, Qatar; 7 College of Health & Life Sciences, Hamad Bin Khalifa University, Doha P.O. Box 17666, Qatar; 8 School of Biochemistry and Immunology, Trinity College Dublin, D02 PN40 Dublin, Ireland; gdavey@tcd.ie

**Keywords:** N-lactoyl phenylalanine, insulin signaling, mitochondrial respiration, cytokines

## Abstract

N-lactoyl amino acids (Lac-AAs) are key players that regulate appetite and body weight. The most prominent and well-studied member is N-lactoyl phenylalanine (Lac-Phe), which can be induced by food intake, exercise and metformin treatment. However, its broader metabolic impact remains insufficiently characterized. This study investigates the effects of Lac-Phe on insulin signaling, inflammation, and mitochondrial respiration using HepG2 and differentiated C2C12 cell models, as well as isolated rat brain mitochondria and synaptosomes. Our results demonstrate that Lac-Phe significantly impairs insulin-stimulated phosphorylation of key proteins in the insulin signaling pathway, particularly in skeletal muscle cells, indicating disrupted insulin signaling. Additionally, Lac-Phe exposure increases the secretion of pro-inflammatory cytokines in C2C12 skeletal muscle cells and markedly impairs mitochondrial respiration in HepG2 liver cells and rat brain-derived synaptosomes, but not in isolated mitochondria. These findings highlight potential adverse metabolic effects of Lac-Phe, especially when administered at high concentrations, and underscore the necessity of conducting a comprehensive risk assessment and dose optimization before considering Lac-Phe or related Lac-AAs as therapeutic agents. Our work provides important insights into the molecular liabilities associated with Lac-Phe and calls for further studies to balance its therapeutic promise against possible metabolic risks.

## 1. Introduction

N-lactoyl amino acids (Lac-AAs) are ubiquitous metabolites that are formed by CNDP2-catalyzed reverse proteolysis of lactate and amino acids [1]. This family of metabolites have garnered significant attention due to their involvement in a variety of physiological processes and pathological conditions. While multiple Lac-AAs have been identified, N-lactoyl phenylalanine (Lac-Phe) is the most prominent member and has been frequently used as a surrogate for other Lac-AAs [2,3,4]. Endogenous levels of Lac-AAs can be induced by exercise, food intake, and treatment with biguanides, especially metformin [2,4,5]. Through in vivo experiments, Lac-Phe was shown to reduce food intake and increase weight loss both upon its induction by metformin or when directly injected in the intraperitoneal region of diet-induced obese mice [4,5]. Lac-Phe was also reported to activate several neuronal regions in the hypothalamus and brainstem [6], suggesting that it has the ability to cross the blood–brain barrier. These regions are involved in the regulation of appetite and energy expenditure [7], indicating that Lac-Phe may exert its effects by influencing the gut–brain axis. In humans, high levels of Lac-Phe were associated with weight loss and reduced visceral and subcutaneous abdominal adipose tissues [4]. Furthermore, its role in metformin-mediated weight loss was confirmed by mediation analysis [5]. Collectively, these findings have contributed to the perception that Lac-Phe may hold a significant therapeutic promise for weight reduction.

Despite their therapeutic potential, Lac-Phe and other Lac-AAs are associated with a variety of medical conditions, including metabolic diseases. For example, high levels of Lac-Phe could independently predict the risk of insulin resistance [8]. In line with this, individuals with type 2 diabetes have significantly higher levels of Lac-AAs than those who are non-diabetic or pre-diabetic [2,9,10]. Mendelian Randomization analysis revealed that multiple Lac-AAs may indeed mediate the progression from pre-diabetes to diabetes [11]. Lac-AAs are also associated with mitochondrial dysfunction and positively correlated with disease severity [12]. Moreover, Lac-Phe is elevated in septic shock [3] and has a causal link to rosacea [13], suggesting it has a possible role in inflammatory conditions.

As Lac-Phe is being considered for use as a therapeutic agent due to its appetite-suppressing and anti-obesity effects and given its involvement in the pathophysiology of multiple diseases, it is important to assess not only its benefits but also its potential risks. Additionally, the downstream effects of Lac-AAs at the molecular level are not fully understood. Therefore, we evaluated the effect of Lac-Phe, as a surrogate for other Lac-AAs, on different biological processes in vitro to assess its potential adverse metabolic effects. Two main cell lines were selected. The first one is HepG2, which was used a surrogate for liver cells. Despite being derived from hepatoblastoma, this cell line was selected because it retains several characteristic properties of primary hepatocytes, sometimes behaving closer to the in vivo environment than immortalized cell lines derived from healthy liver cells [14]. This includes the synthesis of key proteins, inflammatory responses, and insulin-induced Akt Ser473 phosphorylation, a hub signaling molecule examined in this study [15,16,17]). The second type of cell is C2C12 which is also widely employed for metabolic research [18] and was differentiated in this study to be used as a surrogate for skeletal muscle cells. In addition, rat-brain derived preparations were used to study neuronal bioenergetics.

Since the anti-obesity effect of Lac-Phe was absent when dosed orally [4], in vivo studies primarily used intraperitoneal injections to evaluate Lac-Phe effect in mice [4,5,19]. An injection of 50 mg/kg Lac-Phe was commonly used in these studies, a dose that leads to the elevation of Lac-Phe blood concentration up to ~170 µM, which is markedly higher than basal endogenous levels or those induced by metformin or exercise [4]. Moreover, Lac-Phe concentration was not measured in tissues after injection, raising concerns about Lac-Phe’s adverse metabolic effects, especially at the time and location of injection or with long-term repeated treatment and tissue storage. Thus, we used supraphysiological concentrations of Lac-Phe to reveal metabolic liabilities that may arise if it is used as an exogenous therapeutic agent. Here, we show that Lac-Phe could potentially disrupt insulin signaling, stimulate inflammatory cytokines, and interfere with cellular respiration, underscoring the need for further studies for risk assessment and dose adjustment.

## 2. Methods

### 2.1. Cell Culture, Differentiation, and Treatment

C2C12 and HepG2 cell lines were maintained at 37 °C and 5% CO_2_ in high-glucose Dulbecco’s Modified Eagle’s Medium (HG DMEM) (#SH30243.02, Hyclone Cytiva, Logan, UT, USA) supplemented with 10% fetal bovine serum (FBS) (#S1810-500, Biowest, Nuaillé, France) and 1% antibiotic-antimycotic (#15240-062, Gibco, Waltham, MA, USA). Cells were passaged twice a week and kept below 80% confluence. To differentiate C2C12 myoblasts, cells were seeded in 24-well plates at a density of 80,000 cells per well in HG DMEM supplemented with 10%FBS and 1% antibiotic-antimycotic. The next day (day 1), the maintenance medium was replaced with HG DMEM supplemented with 2% FBS and 1% antibiotic–antimycotic. Every 3rd day, the 2% FBS medium was refreshed. On days 7–8, most C2C12 cells had differentiated into myotubes, as assessed by light microscopy, and were used for subsequent experiments. The following compounds were used to treat the cells: L-Phenylalanine (#130310250, Thermo Scientific, Waltham, MA, USA), Sodium L-lactate (#71718-10G, Sigma-Aldrich, St. Louis, MO, USA), and N-Lactoyl-Phenylalanine (#HY-150012, MedChemExpress, Princeton, NJ, USA). All treatments were performed in at least three independent replicates. C2C12 cells were treated on day 7–8 of differentiation. HepG2 cells were treated when cells achieved 70–90% confluence.

### 2.2. Insulin Signaling Assessment

To assess insulin signaling, cells were starved for 2 h in FBS-free Roswell Park Memorial Institute (RPMI) medium (#21875-034, Gibco, Waltham, MA, USA). Then, they were treated for 1 h with 1 mM or 0.5 mM of the indicated compounds prepared in starvation media. This was followed by 100 nM insulin stimulation for 10 min (#12585-014, Gibco, Waltham, MA, USA) and sample collection. For protein extraction, phosphate-buffered saline (PBS)-washed cells were scraped in cold cell lysis buffer (#LQ00006JK0K0RR, part#10024042, Bio-Rad, Hercules, CA, USA), supplemented with protease inhibitor (#A32963, Thermo Scientific, Waltham, MA, USA) and phosphatase inhibitor (#A32957, Thermo Scientific, Waltham, MA, USA) cocktails. Cell lysates were incubated on ice for 30 min and then centrifuged at 15,000× *g* for 10 min at 4 °C. Supernatants were separated and used for subsequent experiments. The protein concentration was determined using QuantiPro™ BCA Assay Kit (#QPBCA-1KT, Sigma, St. Louis, MO, USA) according to the manufacturer’s instructions. Samples were normalized to equal concentrations using cell lysis buffer supplemented with protease and phosphatase inhibitors.

Bio-Plex Pro Cell Signaling Akt Panel 8-Plex Assay kit (#LQ00006JK0K0RR, Bio-Rad, Hercules, CA, USA) was used according to the manufacturer’s instructions to detect the following phosphorylated proteins: Akt (Ser473), BAD (Ser136), GSK-3α/β (Ser21/Ser9), IRS-1 (Ser636/Ser639), mTOR (Ser2448), p70S6 kinase (Thr389), PTEN (Ser380), and S6RP (Ser235/Ser236). In brief, 8-plex antibody-conjugated magnetic beads were washed and incubated with protein extracts overnight. The next day, the beads were washed and incubated with biotinylated detection antibody for 30 min. Then, the beads were washed and incubated with streptavidin-phycoerythrin fluorescent reporter for 10 min. Finally, the beads were washed and resuspended in bead resuspension buffer. Data from the final products were acquired using LABScan3D™ machine (One Lambda, Thermo Fisher Scientific, Waltham, MA, USA) and Luminex xPONENT^®^ for FLEXMAP 3D Software (version 4.3, Luminex Corp., Austin, TX, USA).

### 2.3. Measuring Lac-Phe Concentration in Conditioned Media

HepG2 cells (non-treated, treated with 1 mM Lac-Phe, or treated with 1 mM metformin (#ab146725, Abcam, Cambridge, UK) were incubated at 37 °C and 5% CO_2_, with an equal volume of culture medium, for the indicated timepoints. The conditioned media were collected and submitted to Hamad Medical Corporation (HMC) for the quantification of Lac-Phe concentration by liquid chromatography–mass spectrometry (LC-MS).

### 2.4. Measuring Inflammatory Cytokines

HepG2 cells and differentiated C2C12 cells were treated with 100 nM insulin and 1 mM of either of the indicated metabolites, or an equivalent volume of vehicle control. The treatments were prepared in cell culture media or differentiation media, for HepG2 and C2C12 cells, respectively. The final total volume of the medium was equal for all treatments. The treated cells were incubated at 37 °C and 5%CO_2_ for 24 h to allow the accumulation of detectable amounts of cytokines in the conditioned media which were collected after that. Human ProcartaPlex Mix&Match 8-plex kit (#PPX-08-MXRWF7P, Invitrogen, Waltham, MA, USA) and Mouse ProcartaPlex Mix&Match 6-plex kit (#PPX-06-MXH6CWJ, Invitrogen, Waltham, MA, USA) were used to measure cytokines secreted by HepG2 and C2C12 cells, respectively. The experiments were conducted according to the manufacturer’s instructions. In brief, antibody-conjugated magnetic beads were washed and incubated with conditioned media for 30 min at room temperature, and then overnight at +4 °C. The next day, the beads were washed and incubated with biotinylated detection antibody for 30 min. Then, the beads were washed and incubated with streptavidin-phycoerythrin fluorescent reporter for 30 min. Finally, the beads were washed and resuspended in reading buffer. Data from the final products were acquired using LABScan3D™ machine (One Lambda, Thermo Fisher Scientific, Waltham, MA, USA) and Luminex xPONENT^®^ for FLEXMAP 3D Software (version 4.3, Luminex Corp., Austin, TX, USA).

### 2.5. Measuring Oxygen Consumption of HepG2 and C2C12 Cells

The oxygen consumption rate was measured in HepG2 and differentiated C2C12 cells by high-resolution respirometry using Oxygraph-2k (Oroboros^®^ Instrument Gmbh Corp, Innsbruck, Austria) following the substrate–uncoupler–inhibitor titration (SUIT) protocol as previously described [20]. After 24 h of 100 nM insulin treatment, HepG2 cells or differentiated C2C12 cells were harvested and transferred to a 2 mL O2k chamber with MiR05 medium. Basal respiration rates were measured immediately. Then, a titration of 1, 2, and 4 mM of indicated metabolites was performed. After that, the proton leak state of uncoupled respiration was assessed by adding oligomycin (2.5 µM), and then maximal uncoupled respiration was determined by following titration of carbonylcyanide-4-(trifluoromethoxy)-phenyl-hydrazone (FCCP). Finally, complex I and complex III were inhibited by the sequential addition of rotenone (0.5 µM) and antimycin A (2.5 µM), respectively, and the residual oxygen consumption rate (ROX) was quantified.

### 2.6. Preparation and Measuring Oxygen Consumption of Synaptosomes and Non-Synaptic Mitochondria

Synaptosomes (4 biological replicates) and non-synaptic mitochondria (3 biological replicates) were prepared as described by Kilbride et al. [21]. Briefly, for each preparation, brains from two female Wistar rats (3–4 months old supplied by the Trinity College Dublin Comparative Medicines Unit, License No. AE19136) were chopped and homogenized followed by centrifugation at 823× *g* for 3 min at 4 °C. The subsequent supernatants were centrifuged at 9148× *g* for 10 min at 4 °C. The pellets were resuspended and ultracentrifuged on a discontinuous Ficoll gradient at 104,200× *g* for 45 min at 4 °C. During the isolation, the samples were maintained in STE buffer (320 mM sucrose, 10 mM Tris, 1 mM EDTA, pH 7.4) and stored on ice. The synaptosomes and non-synaptic mitochondria were extracted from the Ficoll gradient, washed in STE buffer to remove any Ficoll, and protein concentrations were determined with the reference standard of bovine serum albumin.

Synaptosomal oxygen consumption rates were investigated with the use of a 1 mL Clark-type oxygen electrode. Krebs buffer (3 mM KCl, 140 mM NaCl, 25 mM Tris-HCl, 10 mM glucose, 2 mM MgCl_2_, 2 mM CaCl_2_, pH 7.4) was used as the reaction buffer for the experiments. Synaptosomes were incubated in Krebs buffer at 37 °C in the oxygen electrode. The oxygen consumption rates were examined for 10 min ± Lac-Phe (0–3 mM). The oxygen consumption rates were determined on at least three separate preparations of synaptosomes. Non-synaptic mitochondria oxygen consumption rates were assayed in a KCl buffer (120 mM KCl, 1 mM EGTA, 10 mM Tris-HCl, pH 7.4) ± Lac-Phe (0–3 mM) in the presence of 10 mM glutamate/5 mM malate to monitor State 4 NADH-linked respiration, followed by 2 µM rotenone and 20 mM succinate to monitor State 4 FADH-linked respiration.

### 2.7. Statistical Analysis

Shapiro–Wilk test was performed to determine normality. The Brown–Forsythe test was used to assess homogeneity of variance. After ensuring normality and homogeneity, data were analyzed by one-way ANOVA followed by a post hoc Dunnett’s test. For data with unequal variance, Welch’s ANOVA followed by Dunnett’s T3 multiple comparisons test was used. Repeated measures one-way ANOVA was used for the assessment of routine mitochondrial respiration upon treatment dose increments for HepG2 cells and C2C12 myotubes. Statistical analysis was performed using Excel (version 16.100, Microsoft Corp., Redmond, WA, USA), RStudio (version 4.0.3, Posit, Boston, MA, USA), and GraphPad Prism (version 10.1.0, GraphPad Software Inc., Boston, MA, USA). GraphPad Prism was used for visualization. Results were expressed as means ± standard error of the mean (SEM).

## 3. Results

### 3.1. Lac-Phe Disrupts Insulin Signaling in Skeletal Muscle Cells

**Figure 1:**
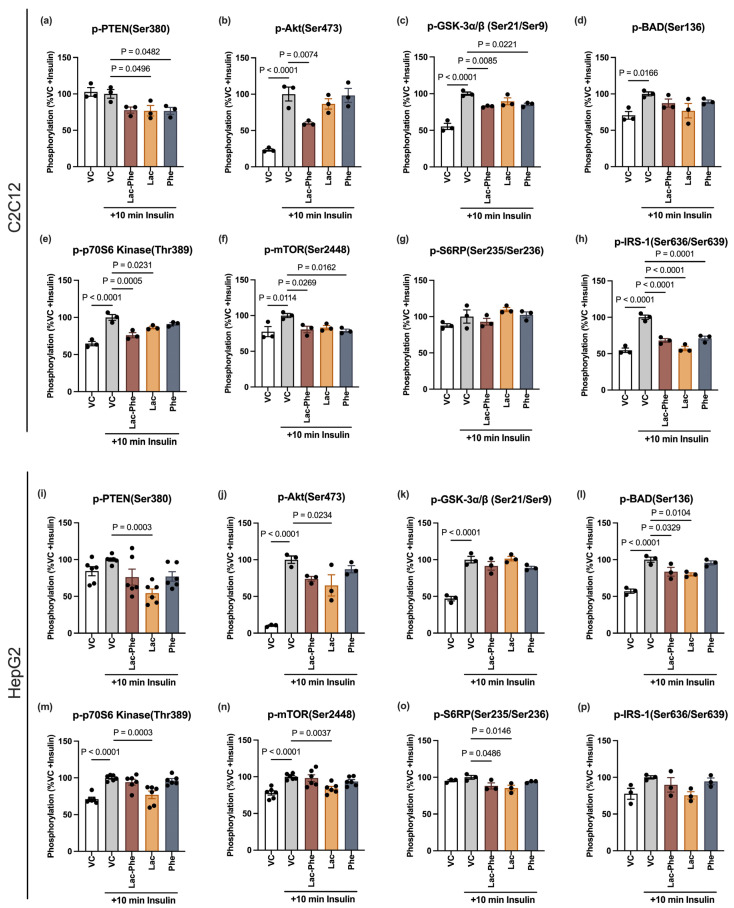
N-lactoyl phenylalanine causes cell type-specific alterations in insulin signaling in C2C12 myotubes and HepG2 cells. Differentiated C2C12 cells (**a**–**h**) and HepG2 cells (**i**–**p**) were starved for 2 h and then treated for 1 h with 1 mM of the indicated metabolites or equivalent volume of vehicle control. This was followed by 10 min 100 nM insulin stimulation where indicated. Phosphorylation levels of the indicated amino acids residues of select proteins along the insulin signaling pathway was assessed. For each protein, all readings were normalized to the insulin-stimulated control (VC + insulin) and all comparisons were made against this condition. Statistical analyses were performed using one-way ANOVA, followed by Dunnett’s test, after ensuring normality and homogeneity of variance. For (**a**–**h**,**j**–**l**,**o**,**p**), *n* = 3/group; for (**i**,**m**,**n**), *n* = 6/group. Error bars are expressed as mean ± SEM. Each dot represents data from an independent biological replicate. Significance levels are indicated with adjusted *p* values. VC: vehicle control; Lac-Phe: N-lactoyl phenylalanine; Lac: lactate; Phe: phenylalanine.

To assess possible risks of Lac-Phe administration considering its association with insulin resistance and type 2 diabetes, the effect of Lac-Phe on insulin signaling was examined in the insulin-responsive cell lines HepG2 (as a surrogate for liver cells) and differentiated C2C12 (as a surrogate for skeletal muscle cells). The differentiation of C2C12 was confirmed visually under a light microscope by observing the formation of myotubes (Supplementary Figure S1). Starved cells were treated for 1 h with vehicle control or 1 mM of either Lac-Phe, lactate or phenylalanine, followed by 10 min insulin stimulation to examine rapid non-genomic changes in the insulin signaling pathway. Successful induction of insulin signaling by insulin treatment was confirmed by comparing insulin-stimulated control cells to cells that were not treated with insulin. Upon insulin stimulation, the phosphorylation of several tested proteins along the insulin signaling pathway was significantly increased, with the biggest effect on p-Akt (Ser473), which serves as a key marker for the activation of this pathway (Figure 1).

Lac-Phe significantly disrupted the insulin signaling pathway in C2C12 myotubes as inferred from reduced insulin-stimulated phosphorylation of Akt at Ser473 (a marker of Akt activation) and its downstream effectors GSK-3α/β (Ser21/Ser9), mTOR (Ser2448), p70S6K (Thr389) and IRS-1 (Ser636/Ser639) (Figure 1b,c,e,f,h). Although lactate and phenylalanine exhibited similar trends, their effects were not consistent and were less pronounced than the effects of Lac-Phe, especially on p-Akt (Ser473) (Dunnett’s multiple comparisons test: VC vs. Lac-Phe, mean difference: 40.00, 95% CI: 11.62 to 68.39, *p* = 0.0074; VC vs. Lac, mean difference: 13.42, 95% CI: −14.96 to 41.81, *p* = 0.4891; VC vs. Phe, mean difference: 1.736, 95% CI: −26.65 to 30.12, *p* = 0.9992) (Figure 1a–h).

While Lac-Phe also suppressed the phosphorylation of Akt at Ser473 in HepG2 cells, its effect did not reach statistical significance (*p* = 0.0947) (Figure 1j). However, it significantly decreased the phosphorylation of BAD (Ser136) and S6RP (Ser235/Ser236) compared to the control, suggesting a possible implication for cell survival and growth (Figure 1l,o). In contrast, Lac-Phe’s precursor, lactate, suppressed insulin-stimulated phosphorylation of multiple proteins along the pathway in HepG2 cells, while phenylalanine had no significant effect on any (Figure 1i–p). Together, the impact of Lac-Phe on insulin signaling might differ depending on the cell type, and Lac-Phe has a similar but not identical signature as compared to its precursors.

To test whether a lower concentration of Lac-Phe would elicit a similar response, cells were treated with 0.5 mM Lac-Phe, lactate, or phenylalanine. Even with a 50% reduction in its concentration, Lac-Phe was still able to inhibit insulin signaling in differentiated C2C12 cells (Supplementary Figure S2a–h). Focusing on Akt as a hub protein, Lac-Phe significantly decreased the insulin-stimulated phosphorylation of Ser473, while lactate and phenylalanine had almost no effect at 0.5 mM (Supplementary Figure S2b). On the other hand, all three metabolites retained their impact on p-IRS-1 (Ser636/Ser639), suggesting potential involvement in the negative feedback mechanism (Supplementary Figure S2h). Conversely, the observed effect of 1 mM Lac-Phe in HepG2 cells was almost completely abolished at a lower concentration (Figure 1l,o; Supplementary Figure S2l,o). A lower concentration of lactate, however, was still able to alter the phosphorylation of multiple proteins along the pathway (Supplementary Figure S2i–p). More detailed statistical results, with ANOVA tables and F ratios, are provided in Supplementary Table S1.

To ensure that the observed effects are due to Lac-Phe and not its degradation in the treatment media, HepG2 cells were treated with 1 mM Lac-Phe for 1 h, 24 h, or 48 h. A sample of conditioned media was taken from each timepoint and tested to determine the Lac-Phe concentration using liquid chromatography–mass spectrometry (LC-MS). As shown in Supplementary Figure S3a, the concentration of Lac-Phe remained close to 1 mM across all tested timepoints in this preliminary observation (*n* = 1), consistent with little to no detectable degradation. In addition, HepG2 cells maintained in culture media without the addition of Lac-Phe have shown accumulation of Lac-Phe in the conditioned media over time, suggesting its endogenous production and secretion using the available nutrients (Supplementary Figure S3b). To support this, HepG2 cells were treated with metformin, which is a known inducer of Lac-Phe production [2,5]. A 48 h treatment with 1 mM Metformin led to further accumulation of Lac-Phe in the conditioned media (Supplementary Figure S3c).

### 3.2. Lac-Phe Increases Pro-Inflammatory Cytokines Secreted from Skeletal Muscle Cells

**Figure 2:**
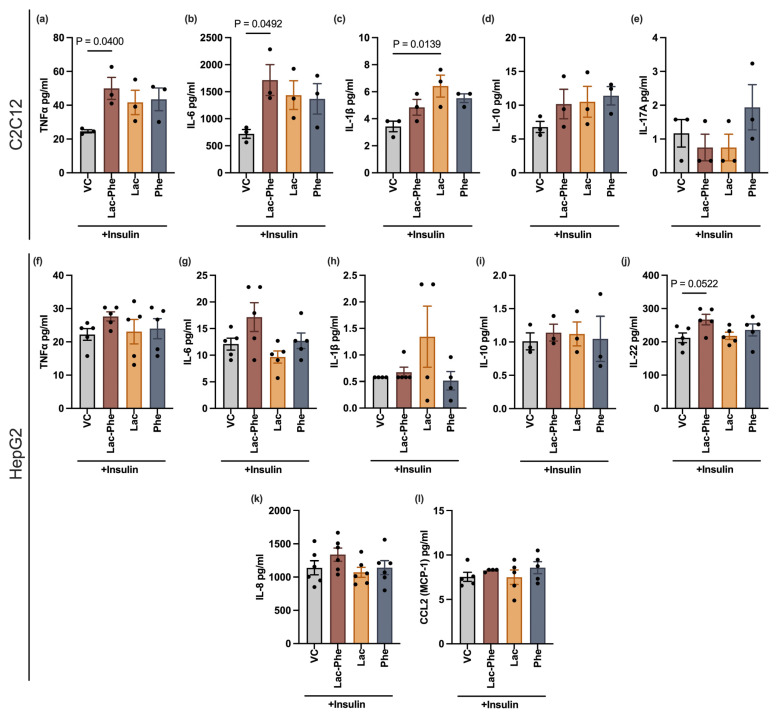
N-lactoyl phenylalanine differentially modulates pro-inflammatory cytokines secreted by C2C12 myotubes and HepG2 cells. Differentiated C2C12 cells (**a**–**e**) and HepG2 cells (**f**–**l**) were treated for 24 h with 100 nM insulin and 1 mM of the indicated metabolites or an equivalent volume of vehicle control. The levels of the indicated cytokines were measured in conditioned media. All comparisons were made against vehicle controls. Statistical analyses of (**a**–**g**,**i**–**l**) were performed using one-way ANOVA, followed by Dunnett’s test, after ensuring normality and homogeneity of variance. Statistical analysis of (**h**) was performed using the Kruskal–Wallis test and Dunn’s multiple comparisons test. For (**a**–**e**,**i**), *n* = 3/group; for (**f**,**g**,**j**), *n* = 5/group; for (**h**), *n* = 4/group except Lac-Phe, which is *n* = 5; for (**k**), *n* = 6/group; for (**l**), *n* = 5/group except Lac-Phe which is *n* = 4. Error bars are expressed as mean ± SEM. Each dot represents data from an independent biological replicate. Significance levels are indicated with adjusted *p* values. VC: vehicle control; Lac-Phe: N-lactoyl phenylalanine; Lac: lactate; Phe: phenylalanine.

Given Lac-Phe’s involvement in inflammatory conditions [3,13] and the connection of inflammation with insulin resistance, it is plausible that Lac-Phe may influence inflammatory signaling. To explore this possibility, the effect of Lac-Phe on a panel of pro-inflammatory markers was examined. HepG2 and differentiated C2C12 cells were treated for 24 h with vehicle control or 1 mM of either Lac-Phe, lactate, or phenylalanine. All treatments were accompanied by insulin to simulate the hyperinsulinemia state in insulin resistance. After 24 h, the levels of a select panel of pro-inflammatory markers were measured in the conditioned media. Compared to cells treated with vehicle control, differentiated C2C12 cells treated with Lac-Phe secreted significantly higher levels of TNF-α and IL-6, but not IL-1β, IL-10, or IL-17A (Figure 2a–e and Supplementary Table S1). Lactate and phenylalanine followed a similar trend but did not reach significance for TNF-α or IL-6 although lactate markedly increased the secretion of IL-1β (Figure 2a–c and Supplementary Table S1). HepG2 cells were influenced by Lac-Phe in the same direction as C2C12 myotubes, but the effect was not significant either (Figure 2f–l and Supplementary Table S1). Additionally, the heatmap analysis of cytokine expression profiles is shown in Supplementary Figure S4. In conclusion, Lac-Phe could stimulate the release of some pro-inflammatory cytokines, and its effect size depends on the cell type.

### 3.3. Lac-Phe Alters the Oxygen Consumption Rates of Liver Cells and Rat Brain-Derived Synaptosomes

**Figure 3:**
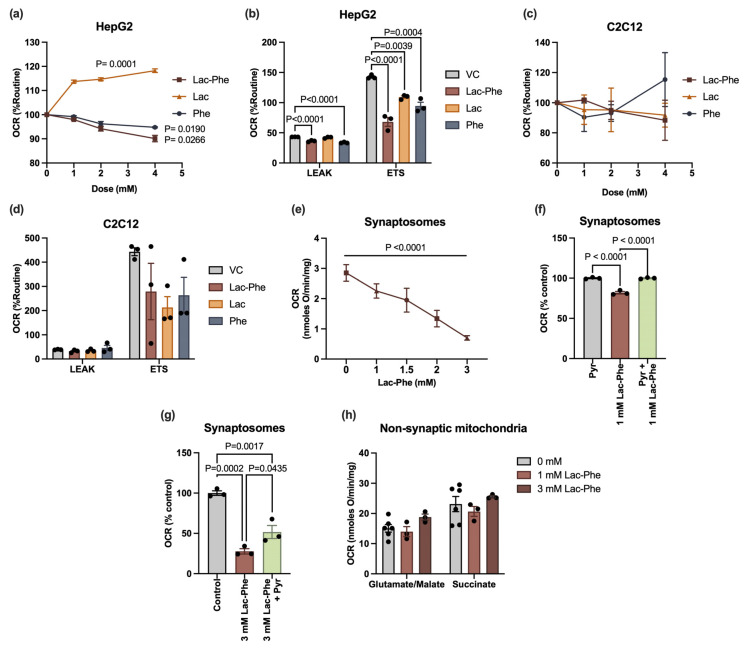
N-lactoyl phenylalanine decreases the oxygen consumption rate of HepG2 cells and rat brain-derived synaptosomes, but not C2C12 myotubes. (**a**) HepG2 cells were treated for 24 h with 100 nM insulin. The oxygen consumption rate (OCR) was measured upon titration of the indicated metabolites or an equivalent volume of vehicle control in an O2k chamber. Readings were normalized to routine OCR and then to the OCR of the vehicle control group for each dose. (**b**) OCR of 24 h 100 nM insulin-treated HepG2 cells was measured after the addition of 2.5 µM oligomycin (LEAK) and FCCP titration (ETS) in the presence of 4 mM Lac-Phe or an equivalent volume of vehicle control in the O2k chamber. Readings were normalized to routine OCR. (**c**,**d**) Differentiated C2C12 cells were treated and assessed as shown in (**a**,**b**), respectively. (**e**) Synaptosomal OCRs were measured in the presence of Lac-Phe (1–3 mM) in Krebs buffer in a Clark oxygen electrode system. (**f**) Five mM pyruvate was added to synaptosomes before addition of 1 mM Lac-Phe. (**g**) Five mM pyruvate was added to synaptosomes following inhibition of OCR with 3 mM Lac-Phe. (**h**) State 4 NADH-linked, and FADH-linked respiration rates were measured in isolated non-synaptic mitochondria in a KCl buffer in the presence of 1 and 3 mM Lac-Phe. Statistical analyses of (**a**,**c**) were performed using repeated measures one-way ANOVA. Statistical analysis of (**e**) was performed using one-way ANOVA after ensuring normality and homogeneity of variance. Statistical analyses of (**b**,**d**,**h**) were performed using one-way ANOVA, followed by Dunnett’s test after ensuring normality and homogeneity of variance. Statistical analyses of (**f**,**g**) were performed using one-way ANOVA, followed by Tukey’s multiple comparisons test after ensuring normality and homogeneity of variance. For (**a**–**d**,**f**,**g**), *n* = 3/group; for (**e**), *n* = 4/group; for (**h**), *n* = 6 for control groups and *n* = 3 for Lac-Phe groups. Error bars are expressed as mean ± SEM. Each dot represents data from an independent biological replicate. Significance levels are indicated with adjusted *p* values. VC: vehicle control; Lac-Phe: N-lactoyl phenylalanine; Lac: lactate; Phe: phenylalanine; LEAK: proton leak state of uncoupled respiration; ETS: maximal uncoupled respiration.

Due to the link between mitochondrial dysfunction and insulin resistance [22], as well as the involvement of Lac-Phe in mitochondrial disease [12], the impact of Lac-Phe on cellular respiration was investigated. HepG2 and differentiated C2C12 cells were treated with insulin for 24 h. Then, their routine respiration was assessed upon in situ treatment with 1–4 mM of Lac-Phe, lactate, or phenylalanine, or an equivalent volume of vehicle control. This was followed by examining the proton leak state of uncoupled respiration (LEAK) and maximal uncoupled respiration (ETS). Lac-Phe treatment of HepG2 cells significantly decreased the basal oxygen consumption rate (OCR_basal_), and this decrease was immediate and dose-dependent (Figure 3a). Whereas phenylalanine similarly decreased OCR_basal_, lactate acted in the opposite direction by increasing OCR_basal_, also in an immediate and dose-dependent manner (Figure 3a). Moreover, Lac-Phe and phenylalanine suppressed LEAK and ETS of HepG2 cells (Figure 3b). Similarly, despite the lactate-mediated enhancement of routine respiration, ETS was significantly lower in lactate-treated cells as compared to control (Figure 3b). On the other hand, Lac-Phe and its precursors did not significantly influence cellular respiration of C2C12 myotubes, neither in the basal state nor in the uncoupled states (Figure 3c,d).

Lastly, given the previously reported effects of Lac-Phe on appetite suppression and activation of brain regions involved in energy homeostasis [3,5,6,7], its potential to modulate neuronal bioenergetics was investigated by assessing its impact on cellular respiration of intact rat brain-derived synaptosomes. The results showed an immediate and dose-dependent inhibition of OCR_basal_ by Lac-Phe (1–3 mM) and this effect was stronger than that observed on HepG2 cells (Figure 3a,e). Additionally, when 5 mM pyruvate was added to synaptosomes before 1 mM Lac-Phe addition, the inhibition of OCR_basal_ was prevented (Figure 3f), suggesting that Lac-Phe is targeting the metabolism upstream of oxidative phosphorylation. The addition of 5 mM pyruvate to synaptosomes following 3 mM Lac-Phe treatment showed partial reversal of inhibition of OCR_basal_ rates (Figure 3g). Additional respirometry data performed on isolated non-synaptic mitochondria (Figure 3h) in the presence of 1- or 3 mM Lac-Phe did not reveal any significant differences in NADH-linked or FADH-linked respiration, ruling out a direct role of Lac-Phe in mitochondrial electron transport chain activity. ANOVA tables and F ratios are provided in Supplementary Table S1.

## 4. Discussion

Lac-Phe has emerged as an endogenous metabolite with advantages for appetite regulation and weight loss [4]. This made it an attractive target for the development of new therapeutic drugs aimed at the treatment of metabolic diseases. However, high Lac-Phe levels have been associated with a variety of disorders, including insulin resistance, inflammatory conditions, and mitochondrial dysfunction [3,8,12,13]. Therefore, while a minimal induction of Lac-Phe by food or exercise may pose a little to no risk, exogenous administration of this metabolite might result in undesirable side effects. The dual nature of Lac-Phe’s pharmacological profile and therapeutic benefits alongside possible metabolic liabilities underscores the importance of comprehensive risk–benefit assessment in its development as a therapeutic agent. Therefore, this study was conducted to determine whether introducing supraphysiological concentrations of Lac-Phe may pose potential adverse metabolic effects.

Lac-Phe significantly interrupted insulin signaling in C2C12 myotubes, including phosphorylated Akt, which is a hub protein affecting several vital targets in the studied pathway [23]. Among these are GSK, BAD, p70S6K and S6RP, suggesting a likely disturbance of glucose homeostasis and cell survival mechanisms as well as cell growth and proliferation [24]. Consistent with this, Lac-Phe was also recently reported to suppress NF-κB signaling [25], another downstream effector of the PI3K-Akt axis [26]. The canonical insulin signaling cascade starts with the autophosphorylation of tyrosine residues in the beta subunits of the insulin receptor upon binding of insulin, leading to the activation of MAPK and PI3K-Akt pathways. Nevertheless, suppression of the phosphorylation of Akt and its downstream targets might not necessarily be due to the suppression of the insulin receptor itself. For example, Lac-Phe might be directly inhibiting insulin receptor’s downstream effectors, such as PDK1 and mTORC2 [23] or other upstream PI3K-independent regulators of Akt, such as ACK1, ATM, and AMPK [27]. The latter is a plausible mechanism, as Lac-Phe was recently reported to directly bind and activate AMPK signaling (40579710).

Moreover, in our study, we observed a trend of insulin-stimulated p-PTEN Ser380 suppression caused by Lac-Phe, although it did not reach statistical significance. Phosphorylation at Ser380, by casein kinase 2, inhibits PTEN’s phosphatase activity, permitting continued activation of the PI3K-Akt pathway [28]. Therefore, Lac-Phe suppression of insulin-stimulated Akt signaling might at least partially be attributed to the direct or indirect activation of PTEN, especially considering the growing evidence of PTEN’s association with the development of insulin resistance and type 2 diabetes [29]. Accordingly, future studies that investigate receptor phosphorylation and upstream regulators, as well as final outcomes, such as glucose uptake, are warranted.

Lac-Phe also decreased p-IRS-1 (Ser636/Ser639), a phosphorylation that is characterized by its inhibitory effect on the insulin signaling pathway [30]. Thus, given that p-Akt (Ser473) is suppressed by Lac-Phe, the decrease in p-IRS-1 (Ser636/Ser639) upon Lac-Phe treatment may seem counterintuitive. However, serine phosphorylation of IRS-1 is part of a negative loop that is initiated upon the activation of mTORC-1-p70S6K axis, which occurs downstream of Akt. Therefore, this observation could be explained by less stimulation of the upstream Akt-mTORC1-p70S6K axis, rather than a direct effect of Lac-Phe on IRS-1, especially that its serine phosphorylation nearly returned to pre-insulin basal levels, but not below [30]. Alternatively, the suppression of p-IRS-1 (Ser636/Ser639) may predict less negative feedback inhibition of insulin signaling upon long-term Lac-Phe treatment. That may hold true considering the previously observed improvement of glucose homeostasis upon chronic but not acute treatment of diet-induced obese mice with Lac-Phe [4,5]. Accordingly, additional research is necessary to assess the effects of Lac-Phe over longer time periods.

A Lac-Phe-mediated increase in the levels of the pro-inflammatory cytokines, TNF-α and IL-6, secreted by skeletal muscle cells may also partially explain the disruption of the insulin signaling pathway. Pro-inflammatory cytokines are known to interfere with insulin signaling [31]. In specific, TNF-α decreases tyrosine phosphorylation and stimulates serine phosphorylation of IRS-1, which may inhibit downstream PI3K/Akt signaling and GLUT4 translocation, eventually reducing glucose uptake [32]. The role of IL-6 is more controversial, despite the association between circulating levels of IL-6 and insulin resistance. IL-6 seems to have opposing effects on different tissue types with apparent benefits for skeletal muscles [33]. Moreover, TNF-α and IL-6 control a wide variety of functions beyond insulin response [33], indicating the possibility of a broader range of physiological consequences of a Lac-Phe-mediated increase in these two cytokines.

Oxygen consumption rates were also tested, since insulin resistance is associated with mitochondrial dysfunction. In line with that, the disruption of the IRS-PI3K-Akt axis can impair mitochondrial biogenesis and OXPHOS [34]. Conversely, mitochondrial dysfunction can alter insulin responses via the accumulation of specific lipid metabolites or ROS generation [22]. Interestingly, while Lac-Phe’s effect on insulin signaling was more pronounced in C2C12 myotubes, inhibition of OCR was only observed in HepG2 cells. Therefore, future targeted experiments utilizing more cell types, timepoints, pathway inhibitors, or genetic models are needed to clarify if impaired insulin signaling occurs upstream, downstream, or independent of respiratory modulation in Lac-Phe-treated cells. Nonetheless, OCR inhibition in HepG2 cells goes in line with the OCR-inhibiting effect of metformin, a known inducer of Lac-Phe.

Moreover, the observed rescue effect of pyruvate on Lac-Phe-mediated inhibition of synaptosomal OCR, added to the lack of effect on isolated mitochondria, suggests that the decrease in OCR is driven by upstream processes such as a decrease in glucose uptake or its conversion to pyruvate. Differential expression of glucose transporters in the three examined cell types may partially explain these cell-type-specific effects. Finally, suppression of ETS in HepG2 cells upon Lac-Phe treatment reflects a possible compromise of metabolic flexibility upon heightened energy requirements, warranting the need to factor in energy demands when assessing the effects of Lac-Phe.

We also show that Lac-Phe precursors, lactate, and phenylalanine had similar effects on the three tested processes, but their profiles were not identical to Lac-Phe. However, the stability of Lac-Phe in conditioned media indicates that the observed effects on the cells are not merely caused by the hydrolysis of Lac-Phe back into its precursors prior to entering the cells. Nevertheless, Lac-Phe might either directly bind its cellular targets, causing these effects, or merely be utilized as a signal transducer that is converted back to lactate and phenylalanine inside the cell. Conversely, lactate and phenylalanine might either simply share targets with Lac-Phe or conjugate into Lac-Phe by CNDP2 inside the cells, resulting in similar effects. Therefore, cell-free studies involving thermodynamics and docking experiments can shed light on the exact mechanisms involved.

Our study has some limitations. For example, supraphysiological concentrations of Lac-Phe were employed in this study to identify acute cellular responses that may not be apparent at lower, transient exposures. However, to recapitulate clinical scenarios, future work should include systematic dose- and time-response analyses to define thresholds for biological effects. Moreover, while the use of HepG2 cells as a surrogate for hepatocytes provides several advantages, the tumorigenic nature of this cell line results in some aberrant processes such as constitutive ERK activation [15] and lower mitochondrial content compared to primary hepatocytes [35]. Therefore, confirming the observed findings in primary hepatocytes is necessary. Furthermore, as 1 h treatment period is unlikely to change the total expression of the examined signaling proteins, only the levels of phosphorylated versions were tested. To confirm that the observed changes are due to altered phosphorylation activity, further studies, especially those involving longer treatment times, will include both total and phosphorylated proteins in parallel. Lastly, this study employed diverse models to assess Lac-Phe’s effects in distinct, metabolically relevant tissues and provide foundational insights into Lac-Phe’s board physiological role. While the differences in responses among the studied models may stem from variations in cell type, they could also be influenced by the species of origin or cell’s ability to cope with the controlled experimental conditions. Therefore, the results should be interpreted with caution, and more studies are required to assess the safety/toxicity of Lac-Phe treatment.

## 5. Conclusions

In conclusion, Lac-Phe may disrupt a variety of cellular processes, potentially contributing to insulin resistance, inflammation, and mitochondrial dysfunction. Therefore, the safety of employing Lac-Phe or other Lac-AAs as exogenous therapeutic agents for the treatment of metabolic diseases requires thorough evaluation of the possible side effects and precise dose adjustment. This study opens new avenues for future functional research into the impact of Lac-Phe and other Lac-AAs at both cellular and systemic scales.

## Data Availability

The datasets used and/or analyzed during the current study are available from the corresponding author on reasonable request.

## Supplementary Materials

The following supporting information can be downloaded at: https://www.mdpi.com/article/10.3390/cells14161296/s1, Figure S1. Differentiation of C2C12 myoblasts into myotubes. C2C12 cell line was differentiated by culturing in high glucose DMEM medium supplemented with 2% fetal bovine serum for 7 days. The figure is a representative light microscopy image showing the formation of elongated, multinucleated myotubes, indicative of successful differentiation. Figure S2. N-lactoyl phenylalanine-mediated suppression of insulin signaling sustains at lower concentration in C2C12 myotubes but not HepG2 cells. Differentiated C2C12 cells (**a**–**h**) and HepG2 cells (**i**–**p**) were starved for 2 h. and then treated for 1 h. with 0.5 mM of the indicated metabolites or an equivalent volume of vehicle control. This was followed by 10 min 100 nM insulin stimulation. Phosphorylation levels of the indicated amino acids residues of select proteins along the insulin signaling pathway was assessed. For each protein, all readings were normalized to the water control and all comparisons were made against this condition. Statistical analyses of (**a**–**l**) and (**n**–**p**) were performed using one-way ANOVA, followed by Dunnett’s test after ensuring normality and homogeneity of variance. Statistical analysis of (m) was performed using Welch’s one-way ANOVA, followed by Dunnet’s T3 multiple comparisons test. For (**a**–**h**,**j**–**l,o,p**), *n* = 3/group; for (**i,m,n**), *n* = 6/group. Error bars are expressed as mean ± SEM. Each dot represents data from an independent biological replicate. Significance levels are indicated with adjusted *p* values. VC: vehicle control; Lac-Phe: N-lactoyl phenylalanine; Lac: lactate; Phe: phenylalanine. Figure S3. Stability and secretion of N-lactoyl phenylalanine in HepG2 conditioned media. Liquid chromatography- mass spectrometry (LC-MS) was used to quantify N-lactoyl phenylalanine (Lac-Phe) in the conditioned media of HepG2 cells which were (**a**) treated with 1 mM N-lactoyl phenylalanine (Lac-Phe) for the indicated timepoints, (**b**) grown in culture media for the indicated timepoints, (**c**) treated with vehicle control (VC) or 1 mM metformin (Met) for 48 h. (*n* = 3). Statistical analyses of (**b**,**c**) were performed using unpaired *t*-test after ensuring normality and homogeneity of variance. For (**a**), *n* = 1/group; for (**b**,**c**), *n* = 3/group. Error bars are expressed as mean ± SEM. Each dot represents data from an independent biological replicate. Significance levels are indicated with exact *p* values. Figure S4. Heatmap analysis of cytokine expression profiles in indicated experimental groups. (**a**) C2C12 myotubes. (**b**) HepG2 cell line. Cells were treated for 24 h. with 100 nM insulin and 1 mM of the indicated metabolites or an equivalent volume of vehicle control. The levels of the indicated cytokines were measured in conditioned media. Columns indicate independent samples. Raws indicate cytokines. VC: vehicle control; Lac-Phe: N-lactoyl phenylalanine; Lac: lactate; Phe: phenylalanine.
