# Supplementary material for: N-Lactoyl Phenylalanine Disrupts Insulin Signaling, Induces Inflammation, and Impairs Mitochondrial Respiration in Cell Models
Source: Cells. 2025 Aug 20;14(16):1296. doi: 10.3390/cells14161296 (PMC12384308; doi:10.3390/cells14161296)
Supplement: Supplementary file 1 [file cells-14-01296-s001.zip › Cells-3789295-proofread_supplementary_file.pdf]

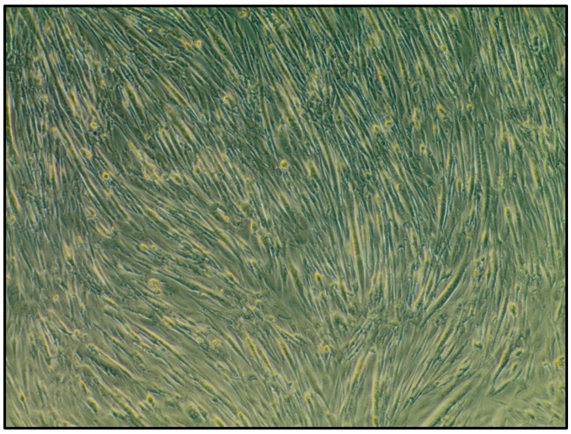

**Supplementary Figure S1. Differentiation of C2C12 myoblasts into myotubes.** C2C12 cell line was differentiated by culturing in high glucose DMEM medium supplemented with 2% fetal bovine serum for 7 days. The figure is a representative light microscopy image showing the formation of elongated, multinucleated myotubes, indicative of successful differentiation.

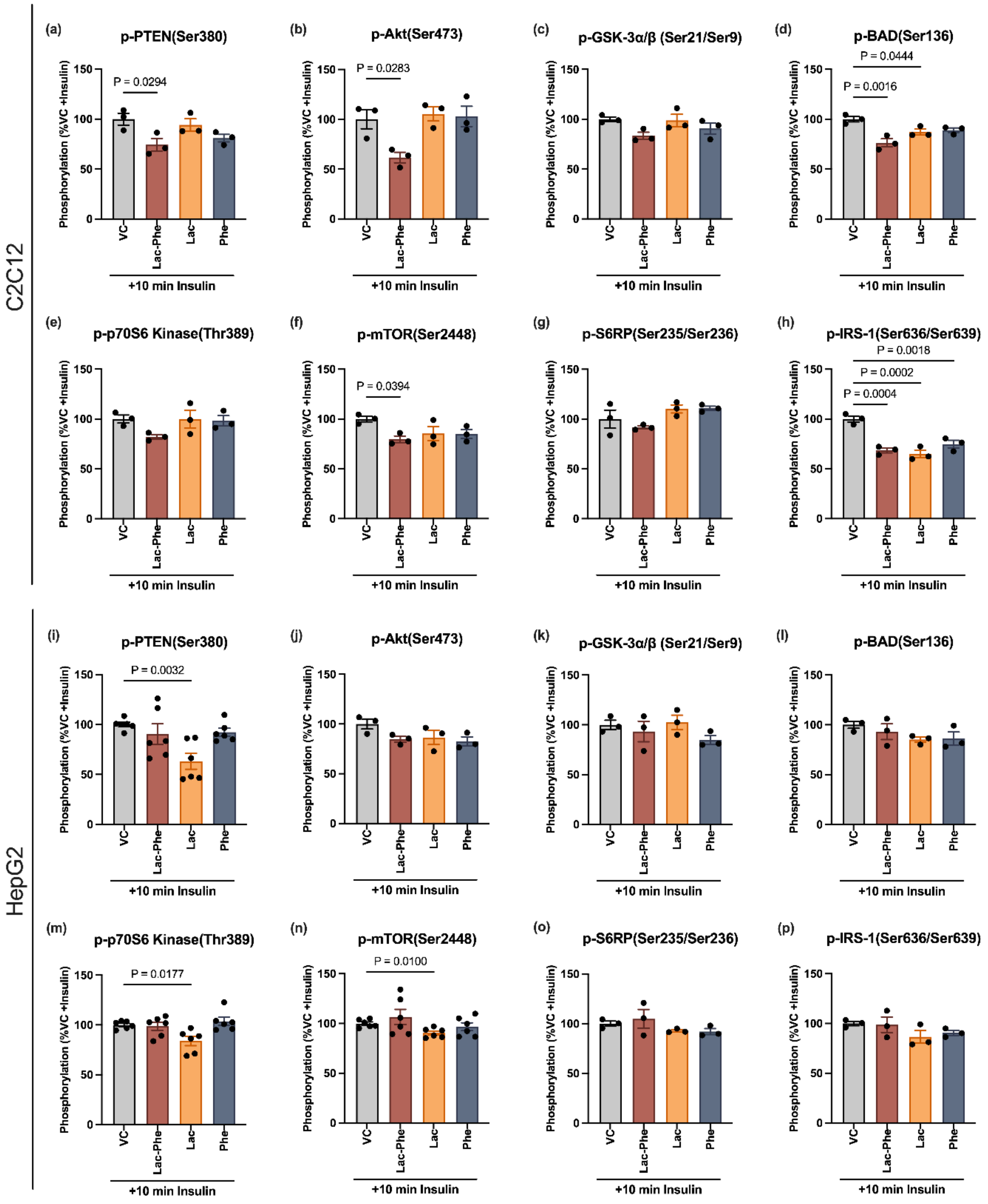


**Supplementary Figure S2. N-lactoyl phenylalanine-mediated suppression of insulin signaling sustains at lower concentration in C2C12 myotubes but not HepG2 cells.** Differentiated C2C12 cells **(a-h)** and HepG2 cells **(i-p)** were starved for 2 hrs. and then treated for 1 hr. with 0.5 mM of the indicated metabolites or an equivalent volume of vehicle control. This was followed by 10 min 100 nM insulin stimulation. Phosphorylation levels of the indicated amino acids residues of select proteins along the insulin signaling pathway was assessed. For each protein, all readings were normalized to the water control and all comparisons were made against this condition. Statistical analyses of (a-l) and (n-p) were performed using one-way ANOVA, followed by Dunnett's test after ensuring normality and homogeneity of variance. Statistical analysis of (m) was performed using Welch's one-way ANOVA, followed by Dunnet's T3

multiple comparisons test. For **(a-h, j-l, o, p)**, *n*=3/ group; for **(i, m, n)**, *n*=6/ group. Error bars are expressed as mean ± SEM. Each dot represents data from an independent biological replicate. Significance levels are indicated with adjusted P values. VC: vehicle control; Lac-Phe: N-lactoyl phenylalanine; Lac: lactate; Phe: phenylalanine.

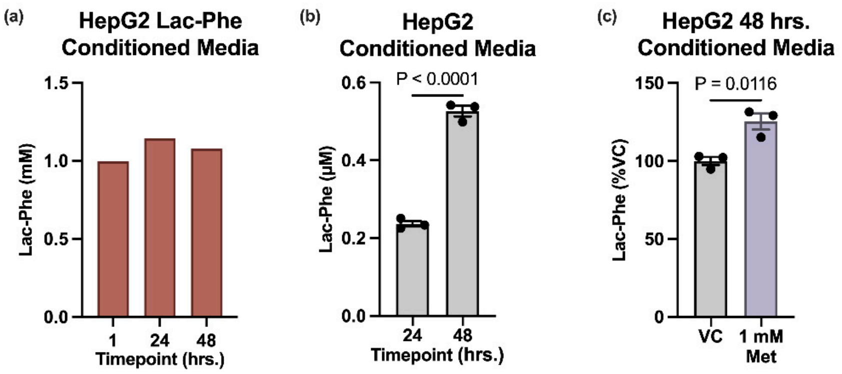


**Supplementary Figure S3. Stability and secretion of N-lactoyl phenylalanine in HepG2 conditioned media.** Liquid chromatography- mass spectrometry (LC-MS) was used to quantify N-lactoyl phenylalanine (Lac-Phe) in the conditioned media of HepG2 cells which were (a) treated with 1 mM N-lactoyl phenylalanine (Lac-Phe) for the indicated timepoints, (b) grown in culture media for the indicated timepoints, (c) treated with vehicle control (VC) or 1 mM metformin (Met) for 48 hrs. (n=3). Statistical analyses of (b, c) were performed using unpaired t-test after ensuring normality and homogeneity of variance. For **(a)**, *n*=1/ group; for **(b, c)**, *n*=3/ group. Error bars are expressed as mean ± SEM. Each dot represents data from an independent biological replicate. Significance levels are indicated with exact P values.

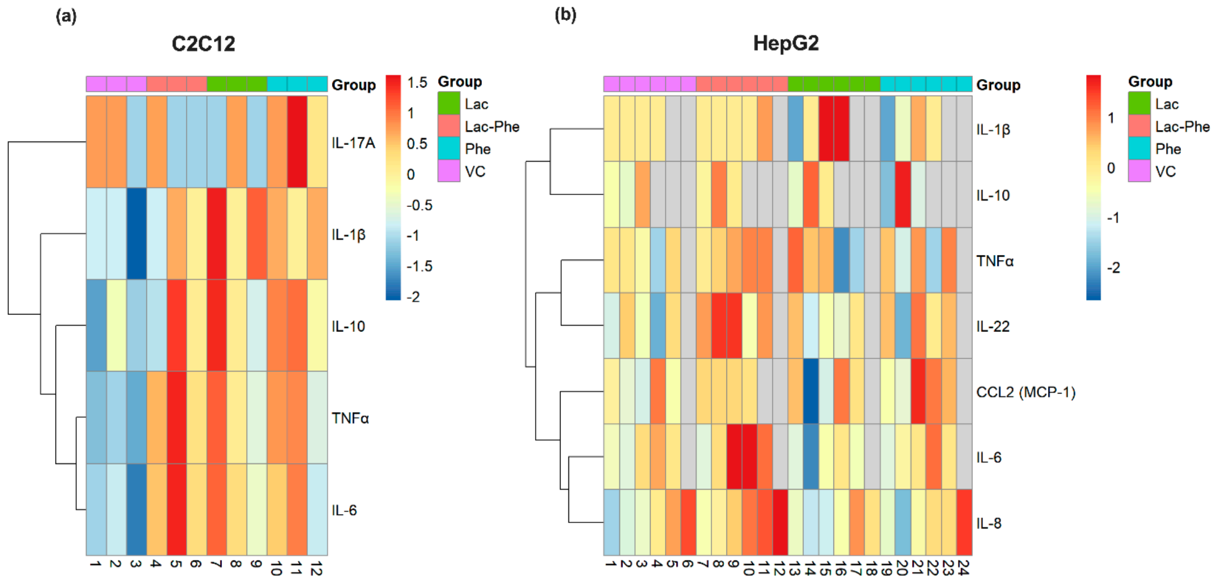


**Supplementary Figure S4. Heatmap analysis of cytokine expression profiles in indicated experimental groups.** (a) C2C12 myotubes. (b) HepG2 cell line. Cells were treated for 24 hrs. with 100 nM insulin and 1 mM of the indicated metabolites or an equivalent volume of vehicle control. The levels of the indicated cytokines were measured in conditioned media. Columns indicate independent samples. Raws indicate cytokines. VC: vehicle control; Lac-Phe: N-lactoyl phenylalanine; Lac: lactate; Phe: phenylalanine.
